# Effect of smoking status and programmed death-ligand 1 expression on the microenvironment and malignant transformation of oral leukoplakia: A retrospective cohort study

**DOI:** 10.1371/journal.pone.0250359

**Published:** 2021-04-16

**Authors:** Takahiro Yagyuu, Naoki Funayama, Mitsuhiko Imada, Tadaaki Kirita

**Affiliations:** Department of Oral and Maxillofacial Surgery, Nara Medical University, Kashihara, Nara, Japan; Queen Mary University of London, UNITED KINGDOM

## Abstract

Tobacco smoking is associated with an increased risk of oral leukoplakia and head and neck cancer. Although it has recently been reported that the establishment of an immunosuppressive microenvironment in oral potentially malignant disorders may lead to malignant transformation, it is unclear whether the microenvironments of oral potentially malignant disorders differ according to smoking status. We examined differences in programmed death-ligand 1 (PD-L1) expression and subepithelial CD163+ TAM and CD8+ cell/lymphocyte counts in the microenvironment of oral leukoplakia of smoking and non-smoking patients and investigated their associations with malignant transformation. Pathology reports and original biopsy request forms from 1995–2015 were retrospectively reviewed. Lesions clinically characterized as white plaques/lesions of the oral mucosa and pathologically diagnosed as oral epithelial dysplasia were included. Immunohistochemistry was performed to evaluate PD-L1 expression and subepithelial CD163+/CD8+ cell counts. The significance of prognostic factors in predicting malignant transformation was determined using Cox regression analysis. Statistical significance was defined as P<0.05. In total, 200 patients with oral leukoplakia were selected. The mean age at diagnosis was higher in non-smoking patients (n = 141; 66.9 years) than in smoking patients (n = 59; 60.5 years). The 5-year cumulative malignant transformation rate was higher in non-smoking patients than in smoking patients (9.3% vs. 3.0%, respectively). Oral leukoplakia was associated with significantly higher PD-L1 expression and increased numbers of subepithelial CD163+ cells in the non-smoking group compared with the smoking group. Non-smoking-related oral leukoplakia with positive PD-L1 expression was associated with a 6.97-fold (95% confidence interval: 2.14–22.7) increased risk of malignant transformation. The microenvironment of oral leukoplakia differed according to smoking status. A combination of smoking status and PD-L1 expression may predict malignant transformation in oral leukoplakia patients. This study highlights the importance of understanding the interaction between smoking and the microenvironment in oral leukoplakia.

## Introduction

Oral potentially malignant disorders (OPMDs) are a group of conditions that may precede the development of oral squamous cell carcinoma (OSCC) [1, 2]. Oral leukoplakia (OL) is the most common OPMD in clinical practice. A systematic review [3] of observational studies of OL reported a malignant transformation rate ranging from 0.13% to 34.0%.

Tobacco smoking is associated with an increased risk of the development of OL and head and neck squamous cell carcinoma (HNSCC) [4–9]. Recently, increased uptake of smoking cessation has been reported [10] in developed countries, such as the United States, Australia, and Japan. A proportional increase in the prevalence of such lesions in non-smokers is expected. The malignant transformation rate of OL is higher in non-smokers than in smokers [11–15]. However, although some recent reports [16, 17] have shown that non-smokers with OSCC may have a worse prognosis, HNSCC is traditionally associated with better overall survival in non-smokers [18]. Non-smoking HNSCC patients had a significantly greater proportion of programmed death-ligand 1 (PD-L1)-positive cells in the microenvironment of HNSCC and had a higher response rate to anti-programmed death 1 (PD-1) checkpoint inhibitors than smoking HNSCC patients [19, 20]. The microenvironment of HNSCC differs according to smoking status. Although it was recently reported [21, 22] that the establishment of an immunosuppressive microenvironment in OPMDs and ductal carcinoma in situ of the breast, mediated by the activation of inhibitory checkpoints (e.g., the PD-1/PD-L1 pathway) or recruitment of immunosuppressive cells (e.g., tumor-associated macrophages [TAMs]), may lead to malignant transformation, it is unclear whether the microenvironment of OPMDs differs according to smoking status.

This study aimed to examine differences in PD-L1 expression and subepithelial CD163+ TAM and CD8+ cell/lymphocyte counts in the OL microenvironment between smoking and non-smoking patients and to investigate whether a combination of smoking status and the expression of such immune markers predicts malignant transformation. This study highlights the importance of understanding the interaction between smoking and the microenvironment in OL.

## Materials and methods

### Sample selection

The pathology reports and original biopsy request forms for all specimens received by the Department of Oral and Maxillofacial Surgery, Nara Medical University, between 1995 and 2015 were retrospectively reviewed. Lesions clinically characterized as white plaques/lesions of the oral mucosa and pathologically diagnosed as having oral epithelial dysplasia (OED) were included. Clinical information including age, sex, smoking and alcohol habits, site of lesion, histopathological details and type of treatment method were retrieved from the patients’ medical records. The exclusion criteria included a previous diagnosis of OSCC/OED, OED with concomitant OSCC at the first visit, insufficient sample for analysis, and insufficient clinical information.

Cigarette equivalents were calculated as follows: one pipe equaled three cigarettes, and one cigar equaled two cigarettes. Smokers were defined as those who had smoked >100 cigarettes (or the equivalent) over the course of their lifetime [23]. Current drinkers were defined as those who had consumed alcoholic beverages of any type within 1 year prior to the date of diagnosis. Former drinkers were defined as those who had never drank or those who had discontinued drinking for >1 year prior to the date of diagnosis [15, 24–26].

This study and all experimental procedures were approved by the ethics committee of Nara Medical University, Nara, Japan (approval date: April 21, 2014; approval number: 827). Informed consent was waived because of the retrospective design, and the analysis used anonymous data. The work was carried out in accordance with The Code of Ethics of the World Medical Association (Declaration of Helsinki).

### Evaluation of OED

OED was graded using hematoxylin and eosin-stained sections without any prior knowledge of the patients’ clinical history. This assessment was based on the World Health Organization (WHO) dysplasia grading presented in 2017, as mild, moderate, and severe dysplasia [1]. For further statistical analyses, OED was classified as low-grade (mild dysplasia) or high-grade (moderate dysplasia and severe dysplasia).

### PD-L1 expression and subepithelial CD163+ and CD8+ cell counts in the microenvironment

PD-L1 expression and subepithelial CD163+ and CD8+ cell counts were evaluated by immunohistochemistry. Paraffin sections at 5 μm thickness were deparaffinized. Antigen retrieval was performed by microwaving slides in citrate buffer (pH 6.0) for 30 minutes. Endogenous peroxidase activity was blocked by 0.3% hydrogen peroxide prepared in methanol for 10 minutes at room temperature. Following several washes with phosphate-buffered saline (PBS), sections were incubated with the following primary antibodies: anti-PD-L1 (1:50 dilution; Spring Bioscience, Pleasanton, CA, USA), anti-CD163 (1:50 dilution; Thermo Fisher Scientific, Kalamazoo, MI, USA), and anti-CD8 (1:100 dilution; Dako, Glostrup, Denmark) for 60 minutes at room temperature. One of three sections of each slide was incubated with isotype IgG antibodies (catalog no. ab172730; Abcam, Tokyo, Japan) to test the specificity of primary antibodies. Sections were washed twice in PBS and subsequently incubated with EnVision FLEX (DAKO JAPAN, Kyoto, Japan) at room temperature for 30 minutes. We visualized the immune reactions by staining slides with 3,3-diaminobenzidine chromogen followed by counterstaining with Mayer’s hematoxylin. The images were captured using an All-in-One Fluorescence BZ-X810 microscope (Keyence, Osaka, Japan).

Membranous PD-L1 staining of atypical epithelium and subepithelial cells was considered to evaluate PD-L1 expression. PD-L1 staining of the atypical epithelium was assessed according to the intensity of the staining and was scored as follows: 0 (negative), 1 (weak to moderate expression), or 2 (strong expression), according to previous reports [22, 27]. PD-L1 staining of subepithelial cells was evaluated by counting PD-L1+ cells in a subepithelial area ≤60 μm from the basement membrane, corresponding to the superficial lamina propria of the oral mucosa. Five high-power fields (40× objective, 10× ocular) were randomly selected, and the average count per mm^2^ was calculated. We subsequently determined an optimal cutoff value for subepithelial PD-L1+ cell counts by receiver operating characteristic curve analysis. Patients with a subepithelial PD-L1+ cell count below the threshold were graded as 0, whereas patients with a subepithelial PD-L1+ cell count above the threshold were graded as 1. Epithelial and subepithelial PD-L1 staining scores (0, 1, or 2, and 0 or 1, respectively) were combined to quantify PD-L1 expression. Final scores ranged from 0 to 3, with PD-L1 positivity defined as a PD-L1 expression score of ≥1.

CD163 is a member of the scavenger receptor cysteine-rich group B family [28] that is exclusively expressed in immunosuppressive TAMs [29–34], whereas CD8 is expressed by cytotoxic T lymphocytes, having a central role in the mediation of anti-tumor immunity [27, 35–38]. CD163+ and CD8+ cells were counted in a subepithelial area ≤60 μm from the basement membrane. Five high-power fields (40× objective, 10× ocular) were randomly selected, and the average count per mm^2^ was calculated.

### Statistical analyses

Student’s t test, chi-squared test, and Mann–Whitney U test were used to determine the significance of associations between smoking status and extracted clinicopathological parameters. Recurrence was defined as the development of OED at the same biopsy site after total resection, including excisional biopsy. Malignancy-free survival was defined as the period from the date of diagnosis to malignant transformation [39]. The 5-year cumulative malignant transformation rate was estimated using the Nelson–Aalen method [40]. Patients without an event were censored at last follow-up.

The independent significance of prognostic factors was determined using the Cox proportional hazards model. The Schoenfeld residual was used to test whether the proportional hazards assumptions were true for any data set. Cox–Snell residuals were used to evaluate the overall fitness of the model [41, 42]. Variables with univariate associations showing P<0.1 were included as confounding factors in the multivariate analysis.

All statistical analyses were conducted using STATA version 15 (StataCorp, College Station, Texas, USA). P<0.05 was considered statistically significant.

## Results

### Clinicopathological characteristics

In total, 200 patients with OL were included; their clinicopathological characteristics are summarized in Table 1. The malignant transformation rate in the entire cohort was 7.5% (n = 15). The mean follow-up period was 55.4 months. One hundred forty-one patients (70.5%) were non-smokers; the remaining 59 patients (29.5%) were smokers. The mean age at diagnosis was higher in non-smoking patients with OL than in smoking patients with OL (66.9 vs. 60.5 years, respectively). The non-smoking group comprised mainly women (73.7% vs. 23.7%, respectively). The proportion of non-drinkers was higher in the non-smoking group than in the smoking group (69.5% vs. 38.9%, respectively). There were no differences in lesion site, treatment course, follow-up period, recurrence, or malignant transformation, according to smoking status.

**Table 1:** Clinical characteristics according to smoking status.

	Total (n = 200)	Non-smoker (n = 141)	Smoker (n = 59)	
Sex				<0.001
Male	82 (41.0%)	37 (26.2%)	45 (76.2%)	
Female	118 (59.0%)	104 (73.7%)	14 (23.7%)	
Age (years)				0.001
Mean ±SD	65.0±12.6	66.9±12.9	60.5±10.6	
Alcohol drinking				<0.001
Never	121 (60.5%)	98 (69.5%)	23 (38.9%)	
Current/ former	79 (39.5%)	43 (30.4%)	36 (61.0%)	
Lesion site				0.11
Tongue	67 (33.5%)	52 (36.8%)	15 (25.4%)	
Other sites	133 (66.5%)	89 (63.1%)	44 (74.5%)	
Course of treatment				0.73
Resection	129 (64.5%)	92 (65.2%)	37 (62.7%)	
Observation	71 (35.5%)	49 (34.7%)	22 (37.2%)	
Follow-up period (months)				0.86
Mean ±SD	55.4±58.6	55.9±57.6	54.3±61.5	
Recurrence after resection				0.72
Yes	22 (17.0%)	15 (16.3%)	7 (18.9%)	
No	107 (82.9%)	77 (83.6%)	30 (81.0%)	
Malignant transformation				0.15
Yes (mean time ±SD, months)	15 (7.5%) (66.5±70.5)	13 (9.2%) (59.8±63.5)	2 (3.3%) (109±130)	
No	185 (92.5%)	128 (90.7%)	57 (96.6%)	

SD, standard deviation.

### OED and PD-L1, CD163, and CD8 expression

Among the 141 patients in the non-smoking group, 93 (65.9%) had low-grade OED and 48 (34.0%) had high-grade OED; among the 59 patients in the smoking group, 39 (66.1%) had low-grade OED and 20 (33.8%) had high-grade OED (Table 2). No significant differences were observed between OED and smoking status.

**Table 2:** Oral epithelial dysplasia and immune markers according to smoking status.

	Total (n = 200)	Non-smoker (n = 141)	Smoker (n = 59)	P-value
Oral epithelial dysplasia				0.98
Low-grade	132 (66.0%)	93 (65.9%)	39 (66.1%)	
High-grade	68 (34.0%)	48 (34.0%)	20 (33.8%)	
PD-L1 expression				0.002
Negative	143 (71.5%)	92 (65.2%)	51 (86.4%)	
Positive	57 (28.5%)	49 (34.7%)	8 (13.5%)	
Subepithelial CD163+ cell count (/mm2)				0.04
Mean ±SD	1026.5±901.8	1110.9±944.6	824.8±760.4	
Subepithelial CD8+ cell count (/mm2)				0.11
Mean ±SD	1184.3±1486.5	1292.0±1615.0	927.1±1091.2	

SD, standard deviation.

**Fig 1:**
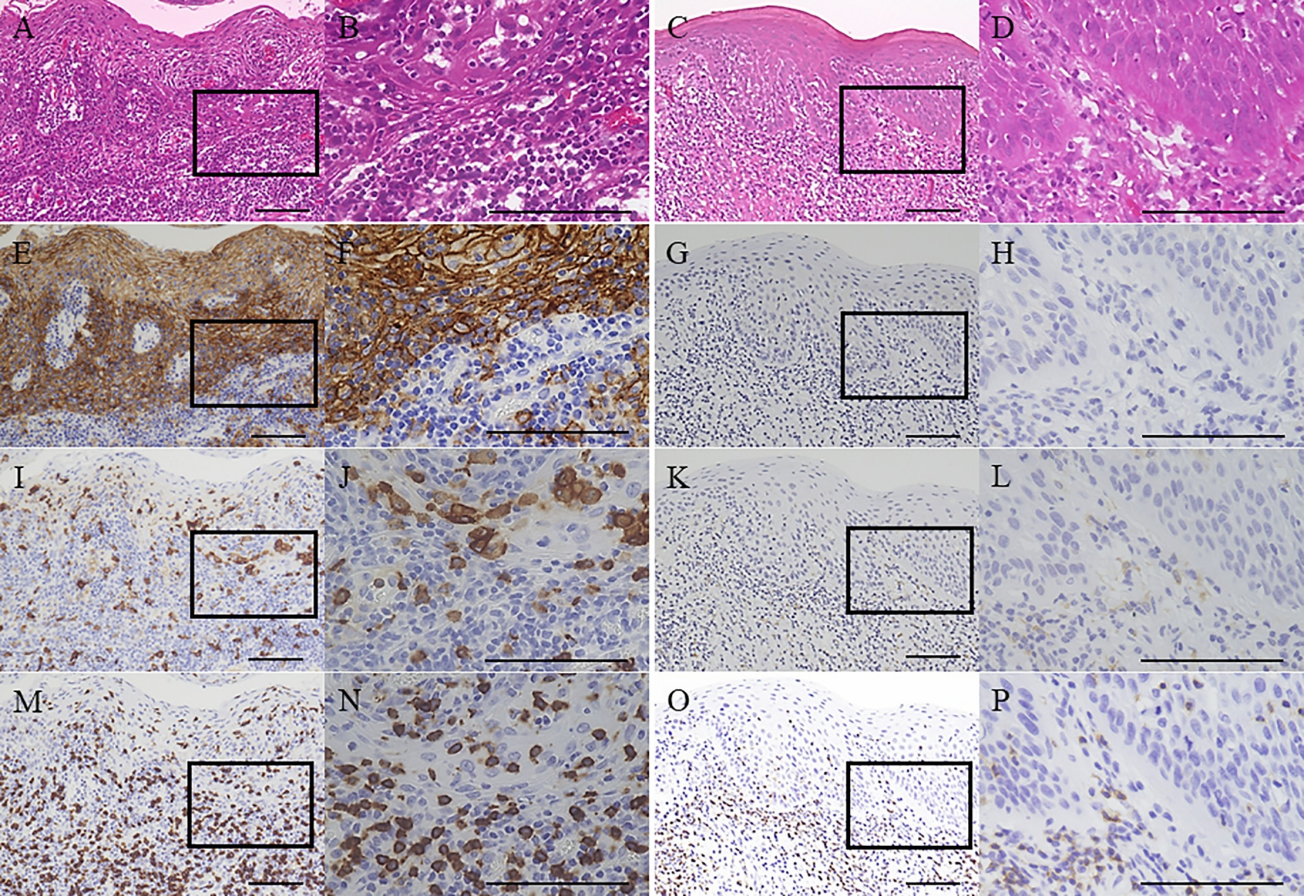
Representative images of immunohistochemical analysis of programmed death-ligand 1 (PD-L1), CD163, and CD8 expression. Hematoxylin and eosin staining of oral leukoplakia (OL) tissue from a non-smoking patient (A, B) and OL tissue from a smoking patient (C, D) showed high-grade oral epithelial dysplasia (OED). PD-L1 staining of OL tissue from a non-smoking patient (E, F) demonstrated strong PD-L1 expression in the overlying epithelium and subepithelial cells. In contrast, PD-L1 staining of OL tissue from a smoking patient (G, H) indicated negative PD-L1 expression in the overlying epithelium and subepithelial cells. CD163 staining of OL tissue from a non-smoking patient (I, J) and OL tissue from a smoking patient (K, L) showed average subepithelial CD163+ cell counts of 3523.3/mm^2^ and 66.6/mm^2^, respectively. CD8 staining of OL tissue from a non-smoking patient (M, N) and OL tissue from a smoking patient (O, P) showed average subepithelial CD8+ cell counts of 4556.0/mm^2^ and 380.0/mm^2^, respectively. Original magnification, ×100 (**A**, C, E, G, I, K, M, O) and ×400 (B, D, F, H, J, L, N, P). Rectangles in (A, C, E, G, I, K, M, O) indicate the areas from where images (B, D, F, H, J, L, N, P) originated. All scale bars denote 100 μm.

Immunohistochemical analysis was performed to investigate PD-L1, CD163, and CD8 expression in the OL microenvironment (Fig 1). Isotype control sections did not show any non-specific staining (S1 Fig).

In the non-smoking group, PD-L1 expression was negative in 92 cases (65.2%) and positive in 49 (34.7%) cases. The mean (±standard deviation) number of subepithelial CD163+ and CD8+ cells was 1110.9±944.6 and 1292.0±1615.0, respectively (S2 Fig). In the smoking group, PD-L1 expression was negative in 51 cases (86.4%) and positive in 8 cases (13.5%). The mean (±standard deviation) number of subepithelial CD163+ and CD8+ cells was 824.8±760.4 and 927.1±1091.2, respectively (S2 Fig). OL was associated with significantly higher PD-L1 expression and increased numbers of subepithelial CD163+ cells in the non-smoking group compared with the smoking group (P = 0.002 and P = 0.04, respectively).

### Five-year cumulative malignant transformation rates

The 5-year cumulative malignant transformation rates of various clinicopathological factors were determined according to smoking status (Table 3). Non-smoking patients with OL had a higher malignancy incidence rate than smoking patients with OL (9.3% vs. 3.0%; 95% confidence interval [CI]: 4.5–18.8 and 0.4–21.5, respectively). A further stratification analysis according to smoking status and extracted clinicopathological characteristics was performed. For almost all subgroups except those with lesion sites other than the tongue, those who underwent resection, and those with subepithelial CD163+ cell counts below the median, the 5-year cumulative malignant transformation rates were higher in non-smoking patients with OL than in smoking patients with OL.

**Table 3:** Five-year cumulative rate of malignant transformation according to smoking status.

	Non-smoker, % (95% CI)	Smoker, % (95% CI)
Overall	9.3 (4.5–18.8)	3.0 (0.4–21.5)
Sex		
Male	19.8 (7.1–55.3)	3.7 (0.5–26.2)
Female	6.0 (2.2–16.3)	0
Age (years)		
≦65	8.6 (2.7–27.2)	4.7 (0.6–33.8)
> 65	9.7 (3.9–24.0)	0
Alcohol drinking		
Never	6.3 (2.3–16.8)	0
Current/former	17.7 (6.2–50.3)	5.2 (0.7–37.3)
Lesion site		
Tongue	18.8 (8.3–42.7)	0
Other sites	4.0 (1.0–16.3)	4.3 (0.6–30.8)
Course of treatment		
Resection	2.2 (0.7–12.5)	4.3 (0.6–30.8)
Observation	25.2 (10.9–58.3)	0
Oral epithelial dysplasia		
Low-grade	2.0 (0.2–14.4)	0
High-grade	21.8 (10.2–46.3)	10.0 (1.4–70.9)
PD-L1 expression		
Negative	4.9 (1.5–16.3)	3.5 (0.5–25.3)
Positive	17.3 (7.1–41.9)	0
Subepithelial CD163+ cell count		
≦ median	0	3.8 (0.5–27.3)
> median	19.3 (9.4–39.6)	0
Subepithelial CD8+ cell count		
≦ median	0.5 (1.2–20.8)	0
> median	13.6 (6.0–30.9)	9.0 (1.2–64.5)

CI, confidence interval.

### Risk factors for malignant transformation according to smoking status

Associations between clinicopathological factors and malignant transformation were evaluated for each subgroup using the Cox proportional hazards model according to smoking status (Table 4). Univariate analysis of non-smoking patients with OL showed that lesion site (tongue), treatment course (observation), high-grade OED, positive PD-L1 expression, and elevated subepithelial CD163+ cell count were significantly associated with malignant transformation. These factors were subsequently included in the multivariate analysis. Non-smoking patients with OL who underwent resection had a statistically significant lower risk of malignant transformation than those managed by observation (hazard ratio [HR], 0.19; 95% CI: 0.05–0.77). High-grade OED and positive PD-L1 expression were also independent risk factors for malignant transformation in non-smoking patients with OL (HR, 2.37 and 5.42; 95% CI: 1.06–5.30 and 1.17–25.0, respectively). Conversely, no significant risk factors were associated with malignant transformation in smoking patients with OL.

**Table 4:** Cox proportional hazards analysis of malignant transformation risk factors according to smoking status.

	Non-smoker				Smoker			
	Univariate		Multivariate		Univariate		Multivariate	
Factors	HR (95% CI)	P-value	HR (95% CI)	P-value	HR (95% CI)	P-value	HR (95% CI)	P-value
Sex (male vs female) *	2.15 (0.68–6.81)	0.19			NA	NA		
Age (>65 vs ≦65) *	0.97 (0.93–1.02)	0.36			1.00 (0.88–1.15)	0.90		
Alcohol consumption (current/ former vs never) *	1.70 (0.65–4.44)	0.27			1.67 (0.11–23.8)	0.70		
Lesion site (tongue vs other sites) *	3.22 (1.04–9.97)	0.04	1.03 (0.27–3.87)	0.95	1.07 (0.05–21.0)	0.96		
Course of treatment (resection vs observation) *	0.32 (0.10–0.98)	0.04	0.19 (0.05–0.77)	0.02	NA	NA		
Oral epithelial dysplasia (high-grade vs low-grade) *	15.9 (2.04–124)	0.008	2.37 (1.06–5.30)	0.03	2.14 (0.13–34.3)	0.59		
PD-L1 expression (positive vs negative) *	5.79 (1.56–21.4)	0.009	5.42 (1.17–25.0)	0.03	NA	NA		
Subepithelial CD163+ cell count	1.00 (1.00–1.001)	0.04	5.69 (0.66–48.4)	0.11	1.00 (0.99–1.003)	0.91		
Subepithelial CD8+ cell count	1.00 (0.99–1.0004)	0.13			0.99 (0.99–1.002)	0.90		

HR, hazard ratio; CI, confidence interval

*, test vs. reference; NA, not applicable.

### Combined effect of smoking status and PD-L1 expression on malignant transformation

**Fig 2:**
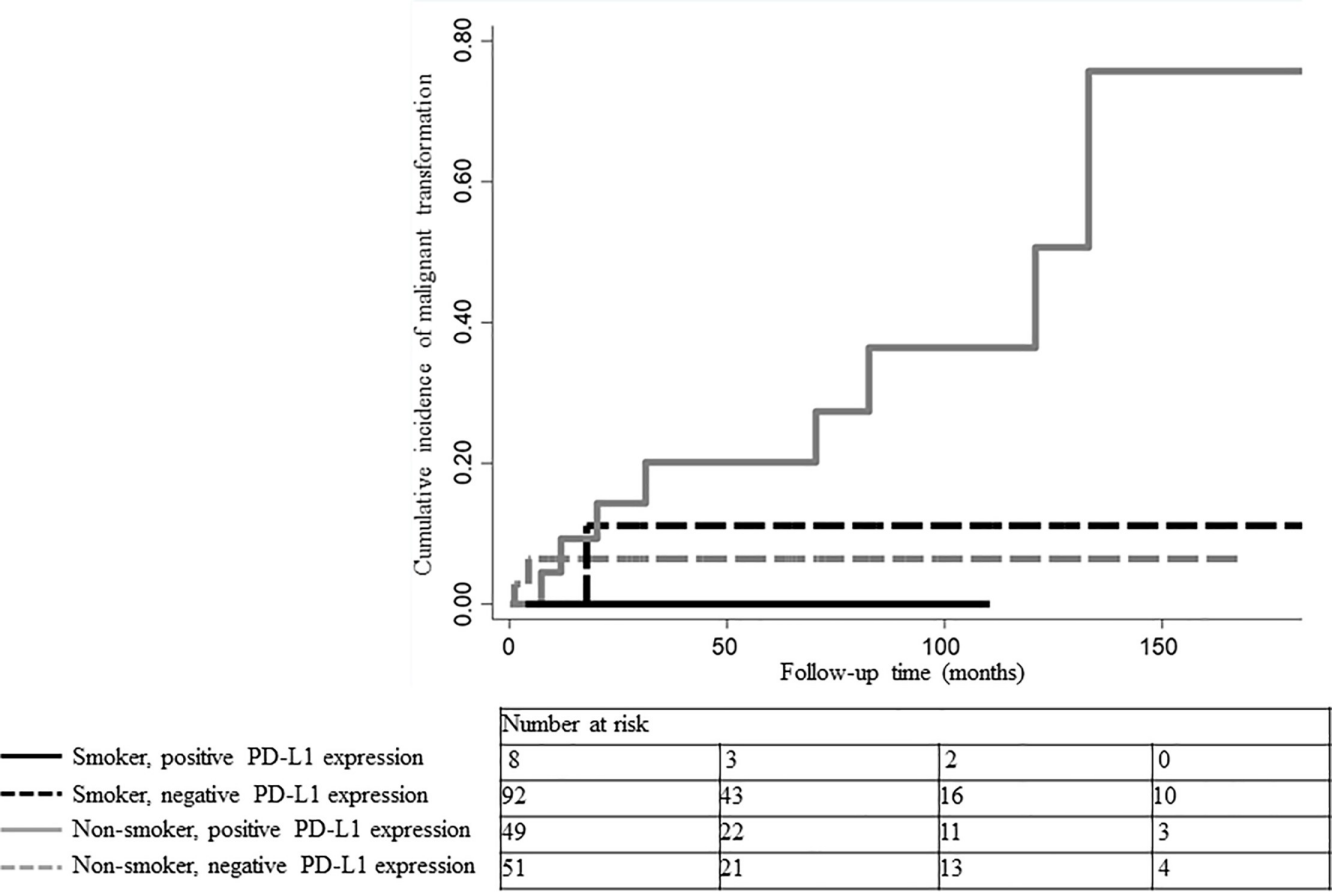
Nelson–Aalen plots of cumulative malignant transformation rates according to smoking status and PD-L1 expression. Smoking patients with positive PD-L1 expression are represented by the solid black line and smoking patients with negative PD-L1 expression by the dashed black line. Non-smoking patients with positive PD-L1 expression are represented by the solid gray line and non-smoking patients with negative PD-L1 expression by the dashed gray line. Non-smoking patients with positive PD-L1 expression had a higher cumulative malignant transformation rate than those in the other three groups.

The combined effect of smoking status and PD-L1 expression on malignant transformation was visualized using Nelson–Aalen plots (Fig 2). Non-smoking patients with positive PD-L1 expression (represented by the solid gray line) had a higher cumulative malignant transformation rate than those in the other three groups. The mean malignant-free survival times and HRs were calculated to assess the combined effect of smoking status and PD-L1 expression on malignant transformation (Table 5). HRs were calculated for the entire cohort. Non-smoking patients with positive PD-L1 expression were associated with a 6.97-fold (95% CI: 2.14–22.7) increased risk of malignant transformation and a significantly shorter time to malignant transformation (mean malignant-free survival time, 137 [95% CI: 109–166] months; P<0.001) than the other groups. Lesions in non-smoking and smoking patients with negative PD-L1 expression were not significantly associated with malignant transformation (P = 0.09 and P = 0.17, respectively). Lesions in smoking patients with positive PD-L1 expression were too few to analyze.

**Table 5:** Combined effect of smoking status and PDL-1 expression on malignant progression.

	Non-smoker		Smoker	
	PD-L1(-)	PD-L1(+)	PD-L1(-)	PD-L1(+)
Mean malignant-free survival time, months (95% CI)	207 (196–218)	137 (109–166)	204 (189–219)	NA
Hazard Ratio (95% CI)	0.39 (0.12–1.24) *	6.97 (2.14–22.7) *	0.38 (0.08–1.77) *	NA*
P-value	0.09*	<0.001*	0.17*	NA*

CI, confidence interval

*, vs. the others in entire cohort; NA, not applicable.

## Discussion

This study examined the clinicopathological characteristics, PD-L1 positivity, and number of subepithelial CD163+ TAMs and CD8+ cells/lymphocytes in the microenvironment of OPMDs according to smoking status. The non-smoking group with OL was 6 years older and had higher proportions of female and non-drinking patients. Although there was no significant difference in OED grade according to smoking status, the 5-year cumulative malignant transformation rate of OL was higher in non-smoking patients than in smoking patients (9.3% vs. 3.0%, respectively). Previous studies [11–15] found that non-smoking patients with OL had different clinical characteristics and a higher risk of malignant transformation than smoking patients with OL. Our study also supports these findings.

Prior to this study, we hypothesized that smoking patients with OL would be more likely to be immunosuppressed than non-smoking patients because chemicals in tobacco smoke cause chronic inflammation in the oral mucosa, which contributes to an immunosuppressive microenvironment. However, beyond our expectations, OL was associated with significantly higher PD-L1 expression and increased numbers of subepithelial CD163+ cells in the non-smoking group compared with the smoking group. Our data showed that the clinical manifestation of OL was delayed by approximately 6 years in non-smoking patients compared with smoking patients. However, once OL occurs in non-smoking patients, an immunosuppressive microenvironment is likely to have already been established through activation of the PD-1/PD-L1 pathway and recruitment of CD163+ TAMs. Furthermore, considering that non-smoking patients with OSCC had a significantly higher proportion of PD-L1-positive cells than smoking patients [19, 20], the PD-1/PD-L1 pathway may be involved in the initiation phase as well as the transformation phase of oral carcinogenesis in non-smoking patients. This retrospective cohort study is one of the first studies to report that patients with OL had a different microenvironment according to smoking status. The elucidation of the mechanism and role of PD-L1 and CD163 expression in OL requires further investigation.

The results of the multivariate Cox regression analysis performed in non-smoking patients showed that PD-L1 expression was significantly associated with malignant transformation (HR, 5.00; 95% CI: 1.02–24.4). We performed a further stratification analysis in the entire cohort to evaluate the combined effect of smoking status and PD-L1 expression on malignant transformation. Lesions in non-smoking patients with positive PD-L1 expression were not only at a significantly elevated risk of malignant transformation, but also progressed to cancer more rapidly. In other words, when OL with PD-L1 positivity is detected in non-smoking patients, it may have already progressed to more advanced stages of oral carcinogenesis than in OL patients with other combinations of smoking status and PD-L1 expression.

The main purpose of identifying OPMDs is to prevent their malignant transformation. The treatment course from careful observation to surgical intervention is usually determined by assessing the malignant potential on the basis of various clinicopathological risk factors, such as size, clinical type (homogeneous vs. heterogeneous), and OED grade. With regard to treatment, some studies [22, 43] suggested that surgery significantly reduced the risk of cancer development. However, other studies [11, 44–46] have shown that surgical interventions do not play a significant role in preventing cancer development. Currently, there are no specific guidelines for the treatment of OPMDs. Our study emphasizes the need for clinicians to consider smoking history and PD-L1 expression in the management of OPMDs. Non-smoking patients with PD-L1-positive lesions may be advised to undergo more aggressive treatment and more intensive follow-up. In addition, immunological approaches to inhibiting the PD-1/PD-L1 pathway may represent a novel treatment modality for preventing the malignant transformation of OPMDs in non-smoking patients.

This study shares the same limitations as previous retrospective cohort studies: selection bias, recall bias, and length of follow-up. Other limitations stem from the self-reported nature of the smoking data and the disregard of the effects of smoking cessation. While our study reported a higher risk of malignant transformation in non-smoking patients with OL than in smoking patients with OL, we have routinely advised smoking patients to cease smoking. However, in this retrospective study, we extracted smoking history at the date of diagnosis and did not consider how many patients may have ceased smoking, that is, the effects of smoking cessation. Smoking cessation results in a reduction in the malignant transformation rates of smoking-related lesions. If a large number of smoking patients had successfully ceased smoking, this could be one of the factors that led to a reduction in the risk of cancer progression in smoking patients with OL. Whether non-smoking patients with OL are really at a higher risk of cancer progression needs to be examined further, along with the effects of smoking cessation, in a future prospective study.

Our study suggests that the risk of malignant transformation is higher in non-smoking patients with OL than in smoking patients with OL (5-year cumulative malignant transformation rate: 9.3% vs. 3.0%, respectively). However, we should not misunderstand the preventative effects of smoking on the malignant transformation of OL. The prevalence of OL is much higher in smoking patients than in non-smoking patients (17.9% vs. 3.9%, respectively) [47–50]. Taken together, our data and these studies suggest that non-smoking patients are less likely to develop OL than smoking patients. However, once OL occurs in non-smoking patients, it is associated with a higher risk of malignant transformation. Tobacco smoking is one of the highest risk factors for oral cancer development, regardless of whether it begins as OL or not [15].

## Conclusions

In non-smoking patients, OL was associated with significantly higher PD-L1 expression and a greater proportion of CD163+ subepithelial cells, which suggests that the OL microenvironment differs according to smoking status. Furthermore, non-smoking OL patients with positive PD-L1 expression were significantly associated with malignant transformation. A combination of smoking status and PD-L1 expression may predict the malignant transformation of OL. This study highlights the importance of understanding the interaction between smoking and the microenvironment in OL.

## Supporting information

S1 Fig Isotype controls for PD-L1, CD163, and CD8 immunohistochemical staining. Isotype control sections for PD-L1 (A), CD163 (B), and CD8 (C) immunohistochemistry did not show any non-specific staining.
(TIF)

S2 Fig Column scatter graphs of subepithelial CD163+ and CD8+ cell counts according to smoking status. The non-smoking group showed significantly increased numbers of subepithelial CD163+ cells compared with the smoking group (P = 0.04) (A). There was no significant difference in subepithelial CD8+ cell count between the non-smoking and smoking groups (P = 0.11) (B).
(TIF)

S1 Table Study data. (XLSX)
