# Supplementary figures and images for: Effect of smoking status and programmed death-ligand 1 expression on the microenvironment and malignant transformation of oral leukoplakia: A retrospective cohort study
Source: PLoS One. 2021 Apr 16;16(4):e0250359. doi: 10.1371/journal.pone.0250359 (PMC8051817; doi:10.1371/journal.pone.0250359)

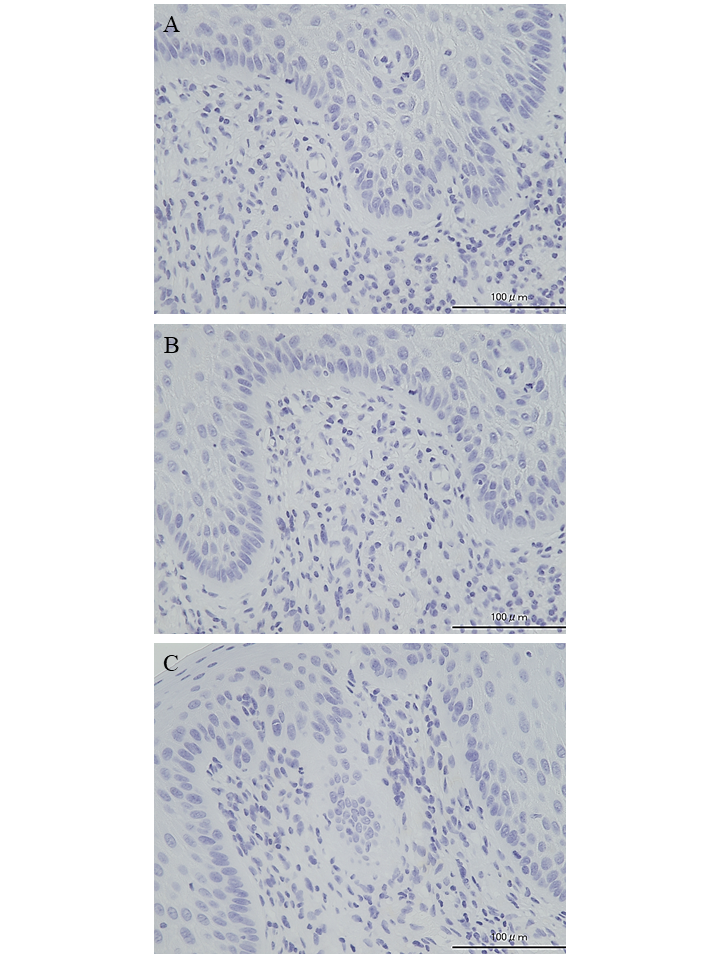

Supplement: S1 Fig — Isotype control sections for PD-L1 (A), CD163 (B), and CD8 (C) immunohistochemistry did not show any non-specific staining. (TIF) [file pone.0250359.s001.tif]

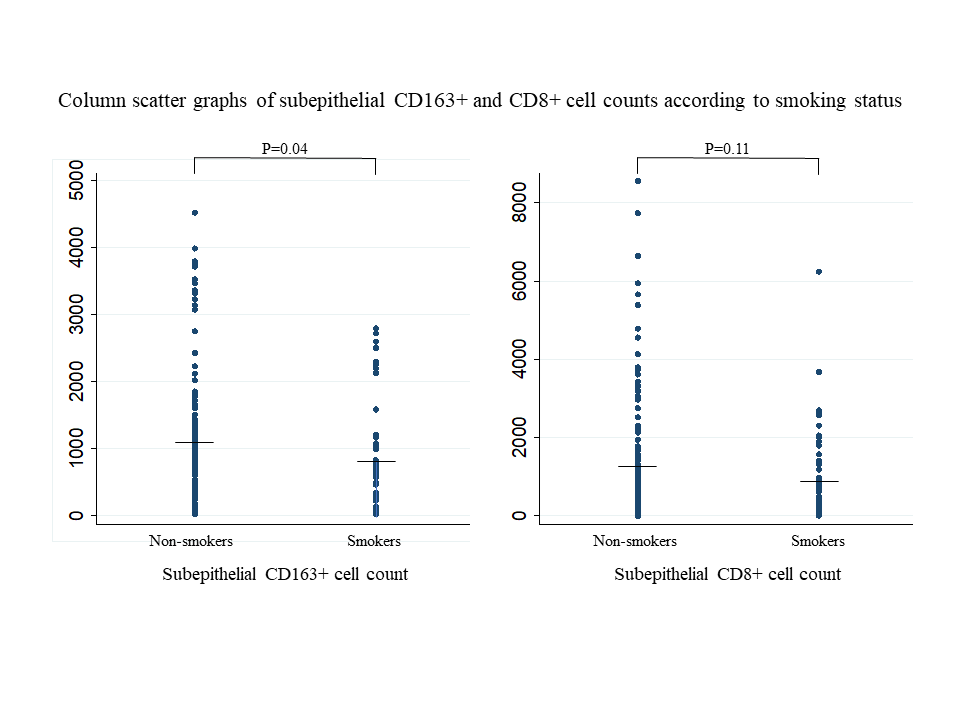

Supplement: S2 Fig — The non-smoking group showed significantly increased numbers of subepithelial CD163+ cells compared with the smoking group (P = 0.04) (A). There was no significant difference in subepithelial CD8+ cell count between the non-smoking and smoking groups (P = 0.11) (B). (TIF) [file pone.0250359.s002.tif]
